# Estrogen Receptor Signalling in Oral Health: From Oral Mucosa to Oral Cancer

**DOI:** 10.31557/APJCP.2026.27.2.411

**Published:** 2026-02-05

**Authors:** Lalit Patil, Ankita Mathur, Snehasish Tripathy, Vini Mehta

**Affiliations:** 1 *Department of Pediatric and Preventive Dentistry, Dr. D. Y. Patil Dental College and Hospital, Dr. D. Y. Patil Vidyapeeth, Pimpri, Pune 411018, India. *; 2 *Dental Research Cell, Dr. D. Y. Patil Dental College and Hospital, Dr. D. Y. Patil Vidyapeeth (deemed to be university), Pimpri, Pune 411018, India. *

**Dear Editor**

Estrogen, a group of steroid hormones produced by the ovaries, testes, and adrenal cortex, are well recognized for their vital roles in a variety of physiological processes, particularly reproductive and sexual functions [1]. Steroid hormones activate two estrogen receptors (ERs): ER alpha (ERα) and ER beta (ERβ), found in diverse tissues including the breast, reproductive organs, and bone. These receptors are found in the nucleus or cytoplasm of normal mucosal cells and have a non-genomic function [2]. They also act as transcription factors, regulating the expression of genes involved in cell growth, differentiation, and viability. 

Pathological ER signaling causes a wide range of illnesses, including metabolic and cardiovascular disease, neurological disorders, inflammatory conditions, osteoporosis, and carcinoma [1]. In cancer, estrogen signalling can influence the progression of cell cycles, apoptotic resistance, and angiogenesis. Abnormal ER signaling is strongly associated with the occurrence and progression of hormone-dependent malignancies, such as breast and ovarian cancer, in which estrogen promotes tumor survival and proliferation. The identification of ERs’ significance in various cancers such as breast cancer has resulted in important breakthroughs in targeted therapy, particularly the creation and use of selective estrogen receptor modulators (SERMs) and aromatase inhibitors, which decrease estrogen synthesis or ER activity in tumor cells [2]. 

*Estrogen Receptors role in Oral mucosal health and oral cancer pathology*

However, the roles of estrogen and its receptors in the oral mucosa have only lately begun to be acknowledged [3]. Immunohistochemical investigations of normal oral mucosa revealed ERβ expression in both males and females [4]. This has been linked to the progression of oral dysplasia and carcinoma. As the oral epithelium undergoes dysplastic alterations, ER expression patterns and functions may shift. Studies have demonstrated that ER expression levels fluctuate as the mucosa progresses from normal to dysplasia [2]. These modifications can have an impact on cell processes such as proliferation rates and oxidative stress response, potentially contributing to dysplasia etiology and development.

In OSCC, estrogen receptors play a significantly more intricate role. The methods by which estrogen signaling influences OSCC are believed to involve both genomic and non-genomic mechanisms [5]. On a genomic level, estrogens may promote cancer development by activating transcription factors that promote tumor cell growth while inhibiting programmed cell death. Non-genomically, ERs can activate several signalling cascades, including the MAPK/ERK, FAK and PI3K/Akt pathways, which promote tumor development and metastasis [6]. The intricacy of these networks highlights the possibility that estrogen signaling influences a variety of cellular activities in OSCC [5]. Studies indicate that [7, 8], overexpression of ERβ in OSCC tissue samples, especially in the cytoplasm and nuclei, has been linked to clinical outcomes including tumor severity and survival rates. Upregulation of ERα in OSCC has been linked to decreased overall survival in male patients and an advanced tumor stage at initial diagnosis [2 ,9].

However, few investigations have suggested that ER signaling may plays a protective effect. Ishida et al. [3] found that ER antagonists have a crucial role in treating human oral SCC by causing cancer cells undergo apoptosis, particularly in areas where ERβ is overexpressed. Similarly, Nelson et al. [10] discovered that high-dose therapy for OSCC using tamoxifen, a non-steroidal anti-estrogen, induces considerable growth inhibition and increases cellular aggregation. Similar finding have been noted in oropharyngeal carcinoma as well. A retrospective study found that ERα is a biomarker for improved overall survival in individuals with HPV+ oropharyngeal squamous cell carcinoma (OPSC) and may aid in improving patient-specific treatments and developing new deintensification medications in such circumstances [11]. Koenigs et al. [12] found that ERα is a biomarker for better survival after chemoradiation in two independent HNSC cohorts. This suggests that ERα could be a therapeutic target for HPV-related OSCC as well due to its role in tumorigenesis and maintenance. 

However, more research into changes in expression patterns and the precise role of estrogen receptor signaling in cell proliferation, invasion, and metastasis in distinct squamous carcinoma cells could have a considerable impact on the landscape of therapy choices for this aggressive malignancy [2]. Given the complexity of ER pathways, identifying specific targets within them could result in more refined and effective treatment drugs. Furthermore, studying how estrogen receptors interact with other molecular pathways in OSCC, such as those involving growth factors or immunological barriers, may uncover synergistic therapy options [6]. Pairing ER modulators and immune checkpoint inhibitors, for example, has the potential to boost anti-tumor immunity while reducing ER-mediated tumor development. Clinical investigations concentrating on ER regulation in OSCC and OPSC are critical for translating these theoretical benefits into clinical reality. To determine the efficacy of ER-targeted medicines, these trials should take into account changes in tumor ER expression as well as stratification by ER status. To address any gender-specific reactions to medication, researchers should investigate the impact of estrogen levels and ER expression in both male and female patients. 

ER-targeted techniques incorporated into the current paradigms of care such as radiation, chemotherapy, and surgery may result in more individualized and efficient care for oral cancer patients. To ensure that all patients benefit from the most recent scientific discoveries, it will be vital to continuously update clinical guidelines as the field advances in order to integrate new information addressing the function of ERs in oral cancer.
